# The Human Antimicrobial Peptides Dermcidin and LL-37 Show Novel Distinct Pathways in Membrane Interactions

**DOI:** 10.3389/fchem.2017.00086

**Published:** 2017-11-07

**Authors:** Kornelius Zeth, Enea Sancho-Vaello

**Affiliations:** ^1^Department of Science and Environment, Roskilde University, Roskilde, Denmark; ^2^Laboratory of Biochemistry, Institut Químic de Sarrià, Universitat Ramon Llull, Barcelona, Spain

**Keywords:** LL-37, structural biology, membranes, artificial, membranes, dermcidins

## Abstract

Mammals protect themselves from inflammation triggered by microorganisms through secretion of antimicrobial peptides (AMPs). One mechanism by which AMPs kill bacterial cells is perforating their membranes. Membrane interactions and pore formation were investigated for α-helical AMPs leading to the formulation of three basic mechanistic models: the barrel stave, toroidal, and carpet model. One major drawback of these models is their simplicity. They do not reflect the real *in vitro* and *in vivo* conditions. To challenge and refine these models using a structure-based approach we set out to investigate how human cathelicidin (LL-37) and dermcidin (DCD) interact with membranes. Both peptides are α-helical and their structures have been solved at atomic resolution. DCD assembles in solution into a hexameric pre-channel complex before the actual membrane targeting and integration step can occur, and the complex follows a deviation of the barrel stave model. LL-37 interacts with lipids and shows the formation of oligomers generating fibril-like supramolecular structures on membranes. LL-37 further assembles into transmembrane pores with yet unknown structure expressing a deviation of the toroidal pore model. Both of their specific targeting mechanisms will be discussed in the context of the “old” models propagated in the literature.

## Human antimicrobial peptides

Antimicrobial peptides evolved during an early stage of the mammalian evolution and represent ancient molecules optimized through their co-evolution with bacteria (Peschel and Sahl, 2006). AMPs are produced by virtually every organism and often comprise the majority of the broad-spectrum antimicrobial activity against fungi, bacteria and viruses. In humans they are an essential part of the innate immune system due to their pleiotropic functions in microbial killing, inflammation, angiogenesis, and wound healing (Nakatsuji and Gallo, 2012). They constantly protect the human body from microbes and inflammation, and their levels can be activated locally and in a timely manner (Zasloff, 2002; Ganz, 2003; Peschel and Sahl, 2006). While the functions of many of these peptides are not well-understood, it has been shown e.g., that a-defensin HD-6 can self-assemble on the bacterial cell surface into nanonets to entangle bacteria (Chu et al., 2012; Ouellette and Selsted, 2012; Chairatana and Nolan, 2017). Dermcidin is a peptide ion channel which can integrate itself into bacterial cytoplasmic membranes to kill bacteria (Song et al., 2013; Zeth, 2013). Pore-like structures can also be formed by granulysin and LL-37 (Anderson et al., 2003; Lee et al., 2011).

In contrast to traditional antibiotics, AMPs often target the bacterial membrane—also known as “the Achilles heel of bacterial cells” (Zasloff, 2002). AMP-membrane interactions are described by three distinct models applicable only to amphipathic α-helical antimicrobial peptides (Zasloff, 2002; Brogden, 2005; Bechinger and Lohner, 2006). All these models are based on the assumption of an initial peptide-lipid interaction mediated through electrostatic properties, followed by free lateral diffusion and pre-assembly of peptides at the membrane surface (Brogden, 2005). The actual membrane insertion step divides the process into three divergent models depending on the particular mode of peptide assembly, the strength of peptide-lipid interactions, and the peptide concentration (Brogden, 2005). The barrel stave model describes the membrane induced assembly of amphipathic peptides into oligomeric transmembrane channels (Baumann and Mueller, 1974). The toroidal model delineates a pore architecture formed by peptide channels laterally stabilized via electrostatic lipid head group interactions (Ludtke et al., 1996; Matsuzaki et al., 1996). Finally, the carpet model describes severe membrane perturbation after the release of mixed peptide-lipid complexes, similar to detergent-induced membrane destruction (Bechinger and Lohner, 2006). To a variable extent, all processes lead to the formation of holes in membranes which—in cytoplasmic membranes—results in the breakdown of the transmembrane potential and cell death (Brogden, 2005). While these three models are frequently used in the literature, recent observations indicate a much greater complexity of AMP-membrane interactions and urge for the development of multistep models developed for each individual AMP. Among the various human AMPs there are two with a clear α-helical secondary structure: dermcidin and LL-37. Our approach aimed for the formulation of refined structure-function-based mechanisms using these peptides, followed by a comparison with the simple standard models.

## Human dermcidin forms a hexameric channel and follows the barrel stave model

Among the major AMPs detected on human skin, dermcidin is enriched as a constitutively expressed peptide (Schittek et al., 2001; Bardan et al., 2004; Paulmann et al., 2012). DCD is active against a broad spectrum of bacteria and fungi at concentrations of ~10 μg/mL (Paulmann et al., 2012). Its antimicrobial activity is robust against changes in pH and ionic strength (Schittek et al., 2001; Paulmann et al., 2012). When isolated from sweat or after recombinant expression, DCD forms an equilibrium mixture of oligomers of varying size, both in solution and in membrane mimetics (Cipáková et al., 2006; Paulmann et al., 2012). Dermcidin is unique amongst AMPs for at least two reasons: it is significantly longer (49 residues) than most of the well-studied AMPs, and its net charge is negative which is in contrast to most of the known AMP molecules reported so far.

**Figure 1:**
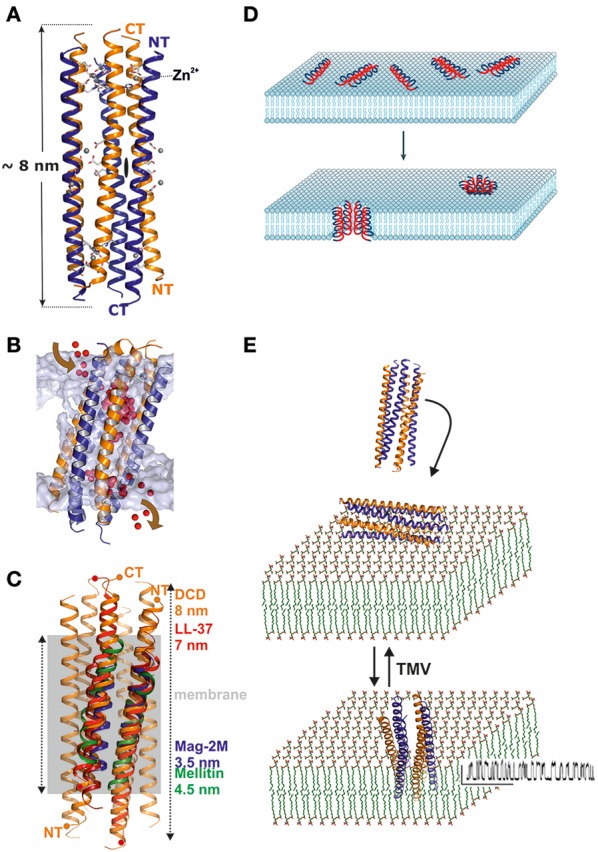
Structure and functional mechanism of human dermcidin. **(A)** Side view of the hexameric structure of dermcidin shown in ribbon representation. The peptide forms regular helixes which are arranged in an anti-parallel manner (highlighted in blue and orange) so that the channel consists of a trimer of dimers which are aligned along the three-fold axis of the channel. The overall length of the channel is 8 nm and zinc binding (Zn^2+^) sites are located at the end of the channel located between two helices. **(B)** Molecular dynamic studies of DCD in artificial membranes demonstrate an unexpected pathway of ion translocation. Ions enter the channel from the side of the membrane at the height of the membrane lipid head groups and leave the channel by the same mechanism. Due to the extension of the channel and the hydrophobic exterior, the energetically most favorable conformation is a tilted channel (20–30° relative to the membrane normal) in the membrane. **(C)** Mechanism of DCD interaction with membranes: the channel exists as a stable hexamer in solution. Interaction of the channel with the membrane does not lead to insertion unless a transmembrane voltage of >100 mV moves the channel into the membrane. Although small, the channel shows clear and defined conductivity steps with a high open probability [see also **(E)**]. **(D)** Simplified mechanism describing the carpet model which explain the activity of AMPs which are in a first step electrostatically attracted by membranes followed by an assembly of peptides and integration into lipid bilayers (Brogden, 2005). This figure is reprinted with permission from Brogden (2005). **(E)** By contrast, dermcidin is already oligomeric in solution and interacts with membranes via electrostatic interactions. Integration of the peptide cannot be detected in biophysical studies unless a transmembrane voltage (TMV) is applied which leads to the detection of a conductive channel (Song et al., 2013).

The structural analysis of DCD provided our group with an unexpected glimpse of a hexameric channel architecture (Song et al., 2013; Figure 1A). Trimers of dimers oriented along the channel axis form the 8 nm extended structure. Each monomer forms two different interfaces to neighboring monomers, one of which is hydrophobic and potentially more stable while the second is hydrophilic. The hexamer and hydrophilic interface formation is stabilized by the presence of divalent ions, in particular zinc ions (Figure 1A; Song et al., 2013). The channel is formed in the absence of lipophilic molecules such as detergents or lipids and is stable with a surplus of hydrophobic residues pointing outwards without being shielded—this is another unique feature of dermcidin (Song et al., 2013). DCD interacts with vesicles e.g., in a planar lipid membrane experiment leading to a channel with an approximate conductance of 100 pS at 1 M KCl but it does not normally insert, unless a voltage of more than 100 mV is applied (Figures 1B,E; Song et al., 2013).

DCD is currently the first antimicrobial peptide discovered at atomic resolution in the channel form (Figures 1A,B). In contrast to the barrel stave model, we show that DCD assembles into this hexameric structure already in solution and subsequently interacts with the bacterial membrane (Song et al., 2013). *In vitro* the channel can be translocated into the membrane by the application of a transmembrane potential. *In vivo* the physiological transmembrane potential formed over the bacterial cytoplasmic membranes may be sufficient to transfer DCD into the membrane. Once inserted in a membrane channel, nanopores destroy the transmembrane potential and this subsequently leads to bacterial cell death (Song et al., 2013). Channel structures such as those of magainin or alamethicin were modeled as oligomers but these models are based on monomeric or dimeric structures assembled on the basis of their transmembrane potential (Figure 1C; Terwilliger and Eisenberg, 1982; Zhu and Shin, 2009; Lorenzón et al., 2012; Hayouka et al., 2013). Their conductance, although defined, is significantly higher (300–600 pS) than for DCD pointing toward the formation of a channel with significantly larger diameter (Figure 1E).

## LL-37 assembles into fiber-like structures as an intermediate step before membrane perforation

LL-37 is an intensively studied peptide with a broad variety of physiological functions, such as in host immunity and antimicrobial activity (Dürr et al., 2006; Vandamme et al., 2012). Its primary sequence clearly indicates amphipathicity, a hallmark of AMPs integrating into biological membranes. Structurally, the peptide was studied using circular dichroism, Fourier transform infrared, and NMR spectroscopy in various media (Johansson et al., 1998; Oren et al., 1999; Li et al., 2006; Wang, 2008). The combined studies indicate that the structure of LL-37 depends on pH, ion strength, and peptide concentrations (Johansson et al., 1998). High resolution studies by NMR were only performed in the presence of 1% SDS, so the structural transition from the solution into a putative membrane associated has not yet been characterized (Wang, 2008).

**Figure 2:**
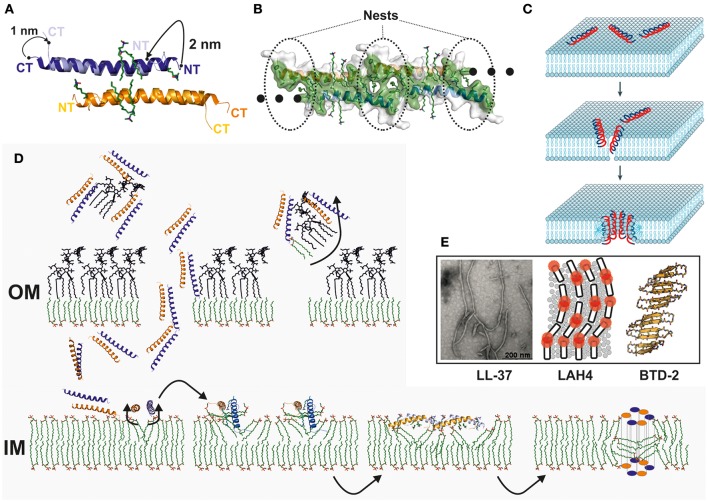
Structure and membrane interaction mechanism of human cathelicidin. **(A)** Structure comparison of the LL-37 dimer in the presence and absence of detergents. Detergents induce a significant conformational change at the N- and C-terminus and discrete detergent binding sites are formed. **(B)** LL-37 tetramer in a surface representation. Hydrophobic residues on the side are marked in green. At the interface between two dimers, nest-like hydrophobic structures are formed to accommodate detergents *in vitro*. Lipid molecules *in vivo* may occupy these detergent positions, and lipid molecules or detergents may initiate the oligomerization of the channel. **(C)** Simplified mechanism describing the toroidal model which explain the activity of AMPs which are in a first step electrostatically attracted by membranes followed by their assembly and partial integration. In a final step the peptides form channels based on peptide-peptide and peptide-lipid interactions after full integration into lipid bilayers (Brogden, 2005). This figure is reprinted with permission from by Brogden (2005). **(D)** Model for the interaction of LL-37 with the cell wall of a Gram-negative bacterium. Significant interactions between LL-37 and LPS have been demonstrated, and, as a hypothesis, LPS may be translocated apart from the cell wall in order to build holes for the translocation of LL-37 into the periplasmic space (Scientific reports in press); Vandamme et al., 2012). Interactions of the peptide with lipid molecules will initiate the conformational changes, and fiber-like oligomers may form on the inner membrane. These fibers lead to an increased local concentration of the peptide and will interfere with the membrane stability. **(E)** Related models and experimental data which were recently published in the literature are based on fluorescence techniques applied to LAH4, crystallography and analysis of crystal packing of BTD-2 and electron microscopy of LL-37 mixed with lipid vesicles (Aisenbrey and Bechinger, 2014; Shahmiri et al., 2016; Wang et al., 2016). LL-37 TEM figure is reprinted from Shahmiri et al. (2016) published in open-access under CC BY 4.0 license. LAH4 figure is reprinted with permission from by Aisenbrey and Bechinger (2014). Copyright 2014 American Chemical Society. BTD-2 figure is reprinted with permission from Wang et al. (2016). Copyright 2016 American Chemical Society.

Because of the obvious lack of reliable experimental data, we crystallized the peptide in the presence and absence of detergents and achieved several structural states (Scientific reports in press). In the absence of detergents, LL-37 forms an anti-parallel dimer similar to the structure of magainin, mellitin, or the antiparallel dimer of DCD (Figure 2A; Terwilliger and Eisenberg, 1982; Hayouka et al., 2013; Song et al., 2013). One of the sides of this dimer is strongly hydrophobic while the opposite side is positively charged. Crystallization in the presence of detergents leads to the reorganization of the dimer, exposing aromatic residues for detergent interactions, and the formation of discrete peptide-detergent complexes (Figures 1A,B; Scientific reports in press). Detergents can bind at the N-terminal region and at the center of the dimeric peptide. Six detergent binding sites are observed per dimer, indicating potential lipid binding sites in the presence of natural or artificial membranes. N-terminally located detergents between two dimers are enclosed by nest-like architecture primarily lined up by aromatic residues (Scientific reports in press; Figure 2B). Furthermore, in the detergent-induced state the molecule forms unidimensional fiber-like chains in the crystal lattice (Figure 2B). These fiber-like structures could also be detected on vesicles using gold-labeled LL-37 and electron microscopy as imaging technique (Scientific reports in press). LL-37 has previously been shown to restructure lipid vesicles into elongated structures, possibly based on the formation of a similar supramolecular structure (Shahmiri et al., 2016). The formation of such fiber-like structures has been described previously for the synthetic peptides LAH4 and BTD-2 (Aisenbrey and Bechinger, 2014; Wang et al., 2016; Figure 2E).

Which model mechanism comes closest to the most recent (Figure 2C) LL-37 data? In the first step, LL-37 interacts with LPS and LTA and possibly removes part of these molecules from the cell wall (Neville et al., 2006). In the second step, according to our own data and the data of others implies that LL-37 can specifically interact with membranes or even specifically with individual lipid head groups via a multi-step mechanism (Scientific reports in press; Shahmiri et al., 2016; Figure 2D). This mechanism is more complicated than the simple toroidal model, where the monomeric peptide assembles on the membrane to form holes on lipid-peptide complexes (Ludtke et al., 1996; Matsuzaki et al., 1996). Oligomeric, fiber-like structures are possibly one intermediate state after potential lipid binding interactions are expressed. These interactions likely destabilize membranes and may also lead to the extraction of lipids from the outer membrane leaflet of the inner membrane. Finally, LL-37 forms channels or pores in membranes to destroy the transmembrane potential but it is unknown if these channels express a peptide-lipid stabilized architecture (Lee et al., 2011; Figure 2D).

## Summary

The growing number of AMPs from many sources forms a solid basis for the development of new antibiotics. This process can be enhanced once their individual mechanisms of action are understood in more detail. Here we show that for two human AMPs their membrane interactions are sophisticated multi-step pathways which deviate from the three simple model mechanisms. Although, our own work has mainly delivered indirect insights based on AMP interactions with detergents and lipids it creates significant improvement of our understanding how DCD and LL-37 target artificial membranes. Together these data represent one critical step forward toward their full mechanistic understanding. However, there is no doubt that true mechanisms *in vivo* in the context of bacterial cell walls are even more complex, and future work needs to initiate studies on the direct interactions of AMPs with the bacterial cell.

## Open questions

DCD and LL-37 are only two selected examples of AMPs, and such do not represent the broadness of mechanisms of how AMPs perturb bacterial membranes. In spite of their improved understanding, general questions remain unanswered e.g.:

- Why has the long DCD channel version, with physical dimensions significantly longer than required for spanning an average membrane thickness been retained?
- How a negatively charged peptide like DCD would interact with an outermost LPS or LTA leaflet layer and how would it pass this layer?
- What is the mechanism by which DCD is translocated over the cell wall of Gram-negative bacteria?

LL-37 activity and killing mechanisms also harbors many secrets e.g.:

- How this peptide interacts with LPS and LTA, and if these molecules are extracted from the membrane to gain access to the cell?
- What is the reason for fiber formation of LL-37 and other AMPs on artificial membranes, and are these fibers also formed on natural membranes?
- Finally, it will be important to test if the detergent binding sites we see in our structures actually resemble lipid binding sites *in vivo*.
- The ultimate step of LL-37 forming pores in membranes and the putative involvement of lipids remains to be shown.

## Author contributions

All authors listed have made a substantial, direct, and intellectual contribution to the work, and approved it for publication.

### Conflict of interest statement

The authors declare that the research was conducted in the absence of any commercial or financial relationships that could be construed as a potential conflict of interest.
